# Nursing research priorities based on CINAHL database: A scoping review

**DOI:** 10.1002/nop2.428

**Published:** 2019-12-26

**Authors:** Hanna Hopia, Johanna Heikkilä

**Affiliations:** ^1^ School of Health and Social Studies JAMK University of Applied Sciences Jyvaskyla Finland; ^2^ School of Health and Social Studies JAMK University of Applied Sciences, Research and Development Jyvaskyla Finland

**Keywords:** clinical nursing research, nursing research, nursing research priorities, scoping review, thematic analyses

## Abstract

**Aim:**

To analyse nursing research based on the CINAHL database to identify research priorities for nursing.

**Design:**

A scoping literature review was conducted. The CINAHL Plus (EBSCO) Full Text was searched between 2012–2018.

**Methods:**

Out of 1522 original publications, 91 fulfilled the inclusion criteria. The Joanna Briggs Institute critical appraisal tools were applied. Data were analysed by a thematic analysis method.

**Results:**

A strong emphasis should be put on development and evaluation of nursing theories and, in addition, randomized controlled trial studies, meta‐synthesis, experimental and intervention studies are needed in nursing research. Development of competencies and skills in the nursing profession ought to be studied more extensively and research should be focused on variety fields of nursing practice.

## 1 INTRODUCTION

Building a research strategy for nursing is vitally important to give the necessary direction for the future. Strategic planning enables us to examine the forthcoming nursing and healthcare research in a systematic way and find areas that need to be further studied. Moreover, nursing strategies help us to implement the necessary procedures and actions to obtain that future. (National Institute of Nursing Research [NINR], 2016, pp. 3–9.) To develop a current, relevant and applicable nursing research strategy, we need to know what the nursing research priorities are locally, nationally and internationally. However, identifying nursing research priorities on different levels can be challenging.

The significance of this review is threefold. First, there seems to be a lack of reviews synthesizing nursing research priorities on a general level. Therefore, it is essential to carry out a scoping review of the literature, which describes current research topics covering different sectors in nursing. Second, the CINAHL database is claimed to be an extremely important electronic database for nurses and nurse researchers (CINAHL databases, 2019). Therefore, it is crucial to summarize the literature on nursing research priorities available in the named database. Third, research priorities are quite often recognized by applying the Delphi technique method. However, broader reviews are also required since they give a wider standpoint by analysing and reflecting the priority areas overall.

## 2 BACKGROUND

Nursing research foundations, organizations, societies and networks throughout the world have reported and updated different research priority areas. The European Nursing Research Foundation (ENRF, n.d.) has, for example, published a strategic and operational research plan for the years 2017–2020 where priority areas of research are defined as follows: positive nursing practice environment, self‐care management, technological health innovations and nursing education. An American counterpart, the National Institute of Nursing Research (NINR, 2016), has also released a strategic plan of nursing research where areas of scientific focus are categorized into these four areas: symptom science to develop personalized strategies, promoting health and preventing illness, improving self‐management strategies for people with chronic diseases and end‐of‐life and palliative care. In addition, many national nurses’ associations and nursing foundations as well as governments have released plans with priorities for conducting and supporting future nursing science. For instance, the American Nurse Association published a strategic plan for the years 2017–2020 (American Nurses Association [ANA], n.d.) and the Scottish government released the “Vision for nursing in Scotland 2030” in 2017 (Nursing, 2017 Vision, 2017). The National League for Nursing (NLN), which is an American organization for nurse faculty and leaders in nursing education, launched the Nursing Education Research Priorities for the years 2016–2019 to promote the role of nurses as scientists (NLN Vision Series, 2016).

In addition to the previously described reports and strategy plans, several studies on research priorities in different fields of nursing have been published in scientific databases. Many of these studies have applied the Delphi technique with a varying number of survey rounds to define the priorities (Cowman et al., 2012; Wynaden et al., 2014). Furthermore, the topics of the research priority studies vary from pressure injuries (Haesler, Carville, & Haesler, 2018) to children's nursing (Brenner et al., 2014) and mental health nursing (Wynaden et al., 2014). Despite the considerable number of research priority publications, limited efforts have been made to understand the big picture of nursing research priorities worldwide and, thus, to describe the essential topics of current research in nursing. Therefore, this review aims to give a synthesis of current identified nursing research priorities by applying a scoping review.

## 3 THE REVIEW

### 3.1 Aim

The aim of this review was to analyse nursing research to identify global research priorities for nursing and its future directions. An additional purpose is to give a synthesis of current nursing research priorities for a future Delphi study. This review also reflects what can be found in CINAHL, one of the largest nursing research databases, about nursing research priorities. The review question addressed was “What are the research priorities for nursing research based on the CINAHL database?”

### 3.2 Design

The scoping review was chosen as a method for its suitability to identify the scope or coverage of a body of literature on a topic (Arksey & O'Malley, 2005). As Peters et al. (2015) have stated, a scoping review is useful when a body of literature has not yet been comprehensively reviewed or exhibits a complex or heterogeneous nature of the evidence. With respect to this review's topic, nursing research priorities, it can be defined as a broad area of interest with a scattered body of literature. In addition, scoping reviews do not aim to produce answers to particular questions but rather to give an overview of the evidence, which is one of the aims of this review. Scoping reviews also have a broader scope than traditional systematic reviews. (Munn et al., 2018; Tricco et al., 2018) This scoping review outlines a preliminary step in the synthesis of the broad literature on nursing research priorities.

### 3.3 Search methods, outcomes and data abstraction

The search of the relevant literature was conducted in the CINAHL Plus (EBSCO) with Full Text (The Cumulative Index to Nursing and Allied Health Literature) database at the end of 2018. The database was selected as it is the largest source for nursing and allied health peer‐reviewed journals and publications in the world and the most used as a nursing research source worldwide (CINAHL databases, 2019). Therefore, no other databases were used for this review. The search was limited between the years 2012–2018 to ensure that the data are rich enough to identify the research priorities in nursing. Since the identification of future strategic priorities is connected to a society at a certain point of time, a longer period might have included topics that have already been studied or are not a current priority. Therefore, a period of 5 years was considered appropriate.

The search strategy was developed using both Medical Subject Headings (MeSH) and free‐text terms likely to appear in the title, abstract or full text of the literature. Search terms were carefully selected to make sure the net was wide enough to include all the relevant publications. The inclusion and exclusion criteria with the search terms are shown in Table 1.

**Table 1:** Inclusion and exclusion criteria and search terms

	Inclusion	Exclusion
Time period	Published 2012–2018	Any publication before 2012
Language	English	Non‐English
Search modes	Boolean/Phrase	Nil
Source types	All	Nil
Study design	Any	Nil
Narrow by subject major	Research priorities	Nil
Limiters	Abstract available	No abstract available
Search terms	(MH "National Institute of Nursing Research (U.S.)") OR (MH "Research Priorities") OR (MH "Research, Nursing") OR (MH "Nurse Researchers") OR (MH "Nursing Administration Research") OR (MH "Education, Nursing, Research‐Based") OR (MH "Clinical Nursing Research") OR (MH "Nursing Practice, Research‐Based") OR (MH "Nursing Care Studies") OR "research priorities AND nursing"	

In total, 1,522 publications were identified by the electronic search. A review team with three experts was formed to conduct the selection strategy. In all the following stages, two of the experts, first independently and then together, reviewed the publications with respect to the aims and research question of the review. The third expert engaged in the critical appraisal phase. As a result of the screening, 1,071 of the publications were excluded. The reason for the exclusion was the unsuitability of the titles about the aim of the literature review. Next, the remaining 451 abstracts were assessed for eligibility and 212 of them were excluded for the following reasons: duplicate article (*N* = 3), full article was not available (*N* = 1) and not suitable for the aim of the review (*N* = 208). Then, two researchers assessed 239 full‐text publications. The discussion was carried out until consensus was reached, and as a result, 143 publications out of 239 were excluded for not being suitable for the aim of this review. Thus, the literature review included 96 publications at this point.

### 3.4 Quality appraisal of the included publications

Even though it is not necessary to conduct the critical appraisal procedure in scoping reviews (Munn et al., 2018; Tricco et al., 2018), the Joanna Briggs Institute's (JBI) Critical appraisal tools (2017) were chosen to determine the methodological quality and general adequacy of the publications included in this review. The JBI checklists were selected as they are intended for nursing and healthcare studies specifically.

In the critical appraisal procedure, two independent reviewers evaluated the 96 full‐text publications for their quality. The design and methodology of each publication were paired with the appropriate JBI checklist, and thus, the following tools were used for the evaluation: analytic cross‐sectional studies, systematic reviews, qualitative studies and text and opinion. The decision about the scoring system and the cut‐off for the inclusion of a publication were made in advance and was agreed on by both participating reviewers before critical appraisal commenced. It was decided that the publication had to fulfil 50% of the assessment criteria and as every JBI checklist has a different assessment item range, the exact cut‐off scores that were applied are illustrated in Table 2. As for Delphi studies, which often use a variety of methods, the tool of analytic cross‐sectional studies was used in most cases. In terms of argumentative, discussion and contemporary issues papers, strategic and policy papers and editorials, the text and opinion checklist were employed.

**Table 2:** Results of the quality appraisal according to number of publications

Critical appraisal checklist	Assessment items range	Cut‐off score	Number of publications assessed (*N* = 91)	Number of excluded publications
Analytical cross‐sectional studies	1–8	4	18	2
Systematic reviews	1–11	6	26	
Qualitative studies	1–10	5	3	1
Text and opinion	1–6	3	44	2

**Figure 1:**
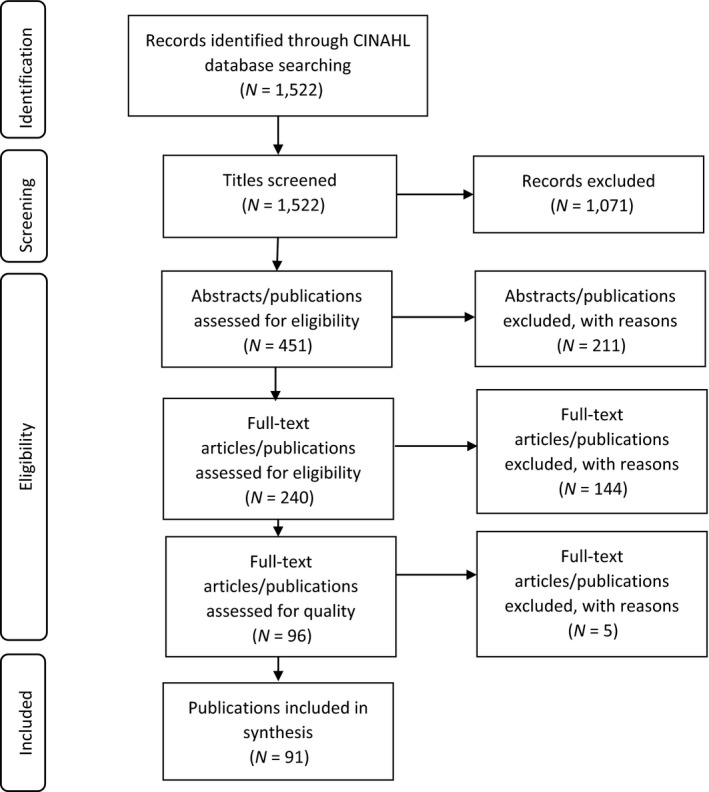
Flow diagram of the literature search on research priorities in nursing from 2012 to 2018

After the independent appraisals, the reviewers discussed the cases where their evaluations differed to reach consensus. Eventually, five publications were excluded from the synthesis for having low quality, leaving 91 articles for the review. Figure 1 shows the flow diagram of the literature search.

### 3.5 Data analysis

The data were analysed by using the thematic analysis described by Braun and Clarke (2006). First, 91 articles were read through and the results or main content from each article were extracted into a table. Furthermore, the types of the included publications were identified and the results are shown in Table 3. Second, codes were generated and clustered into sub‐themes that were related. The theme was defined as a coherent integration of the different pieces of data that constitute the findings. The idea of the theme was that it captured something significant about the data in relation to research question and aim and it represented a pattern or meaning in the data set. After clustering initial codes into sub‐themes, themes were identified, defined and named. Lastly, the key themes were refined with regard to aim and overall meaning of the review. To increase the verification of the analysis, peer debriefing was applied during the coding process. In practice, two members of the research team regularly discussed their personal insight and perception of all the aspects of the analysed data. This helped us to examine how our thoughts and ideas evolved as we engaged more deeply with the analysis process. (Braun & Clarke, 2006; Nowell, Norris, White, & Moules, 2017; Vaismoradi, Turunen, & Bondas, 2013).

**Table 3:** Types of publications

Publication type	Number of publications (*N* = 91)
Systematic and other types of literature review	26
Discussion/contemporary issues article	24
Delphi/consensus‐building study	16
Strategic and policy paper	12
Editorial	5
Empirical quantitative study	5
Empirical qualitative study	3

### 3.6 Ethics

Ethical approval was not required. The review was conducted according to good scientific integrity.

## 4 RESULTS

Almost a third (26) of the selected publications were systematic or other types of literature reviews, and a little over fourth (24) were discussion/contemporary issues articles. The research articles were mostly conducted with Delphi or other consensus‐building methods (16) and a minority with quantitative (5) or qualitative methods (3). The rest of the publications were policy papers (12) and editorials (5) (see Table 3). More than half (53) of the publications originated from the North America (United States 48; Canada 6), 24 articles from Europe, 7 from Australia, 3 from South Africa and single publications from Asian countries Iran, China and Korea.

**Figure 2:**
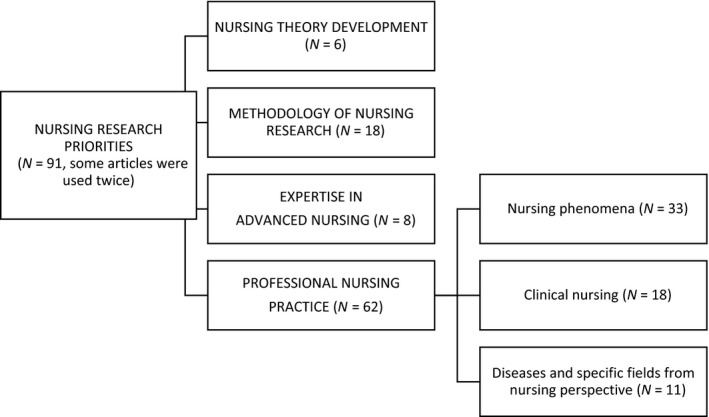
Research priorities of nursing

Four key themes for nursing research were identified. Three of them are related to the following areas: nursing theory development, methodology of nursing research and expertise in advanced nursing. The fourth key theme is professional nursing practice, which is divided into three different domains: (a) nursing phenomena, (b) clinical nursing and (c) diseases and specific fields from the nursing perspective. The number of included publications in each key theme can be seen in Figure 2. The key theme of professional nursing practice covers 62 out of 91 publications while the themes expertise in advanced nursing and nursing theory development include eight and six publications, respectively. In terms of the domain of nursing phenomena, it contains 33 publications whereas clinical nursing and diseases and specific fields from the nursing perspective domains hold 18 and 11 publications. The included publications are separately listed at the end of this article.

### 4.1 Nursing theory development

Several nursing researchers have emphasized that continuing research and in‐depth academic discussion should be carried out in the area of nursing theory development (Im, 2014a; Jairath, Peden‐McAlpine, Sullivan, Vessey, & Henly, 2018; Turkel et al., 2018). In particular, the theories that are strongly based on practice such as situation‐specific theories and middle‐range theories need to be further developed. Furthermore, nursing theories should be evaluated frequently in nursing practice (Im, 2014b.). Researchers (Im, 2014a; Turkel et al., 2018) warn that unless more emphasis is put on theory development and evaluation, theories can silently be erased from nursing science. Jairath et al. (2018) underlined that nursing metaparadigms (health, person, nursing and environment) need revision in the current scientific and practice climates. According to Jairath et al. (2018) and Rolfe (2016), the gap between research and theory is growing and, therefore, building practices based on theories as well as constant evaluation of nursing theories are crucial areas in current nursing science. That is, constant re‐examination of nursing theories is needed to clarify the domain of nursing and to guide nursing practice and science. Some scholars even claim that nursing science is at a critical point in its development and nursing theories should guide nursing practice in terms of individualized holistic care (Turkel et al., 2018). As Kelly, Kent, McMahon, Taylor, and Traynor (2016) argue, attention needs to be focused on how to ensure that the impact of nursing research and research on nursing is captured and celebrated. The impact of nursing research and its role in theory development should be fully recognized now and in future (Kelly et al., 2016).

### 4.2 Methodology of nursing research

Nursing science researchers are of the opinion that more randomized controlled trial (RCT) studies, meta‐synthesis, experimental and intervention studies are needed (Baldi et al., 2014; Richards, Coulthard, & Borglin, 2014). In addition, research that is more robust is required when measuring outcomes of nursing interventions, whereas methodological rigour is warranted when conducting economic evaluations in nursing (Cook, Morrison, Eaton, Theodore, & Doorenbos, 2017). Cook et al. (2017) highlighted that it is critical to show the value of nursing through high‐quality and well‐designed economic evaluation research. More importance should also be attached to the appropriate methods of conducting evidence‐based nursing research (Zhao et al., 2018). Behavioural science such as big data and quantitative science as well as patient‐reported outcomes and health economics are relevant to nursing research, and more stress should be put on these areas and data collection methods (Bates, Saria, Ohno‐Machado, Shah, & Escobar, 2014; Brennan & Bakken, 2015; Henly, McCarthy, Wyman, Alt‐White, et al., 2015a; Henly, McCarthy, Wyman, Heitkemper, et al., 2015b; Westra et al., 2017). It is important to increase the visibility of big data conducted by nurse scientists (Westra et al., 2017). Gephart, Davis, and Shea (2018) stated that to stay contemporary, nurse scientists need to build from both theory and big data in ways that make nursing knowledge more accessible and visible. Overall, nursing researchers should systematically use large health registers of data as well as big data in nursing research. For example, it is crucial to use large health registry data when studying the outcomes of maternity care (Lamminpää, Gissler, & Vehviläinen‐Julkunen, 2017), as well as nursing research of older people (Stolt, Suhonen, Eloranta, Elo, & Isola, 2017). Big data with large data set analysis from the nursing perspective has the potential to give a better understanding of patient phenomena and in tailoring interventions that are personalized to the patient (Brennan & Bakken, 2015).

In terms of implementation science, Curtis, Fry, Shaban, and Considine (2017) placed emphasis on the fact that more attention ought to be paid to how to translate research evidence into clinical nursing practice. Translation should be considered in research design, including an evaluation of the research implementation. New methods are necessary to build knowledge translation into study design and conduct (Curtis et al., 2017; Weiss, Bobay, Johantgen, & Shirey, 2018). Another reason to increase the amount of translation research is to ensure that evidence is implemented into nursing practice based on the latest research (Deutschman, Ahrens, Cairns, Sessler, & Parsons, 2012; Heitkemper et al., 2015; Weiss et al., 2018). Overall, researchers have stated that the impact of nursing research is now hidden, and it must be recognized by conducting scientifically sound studies with generalizable results (Baldi et al., 2014; Kim, Sefcik, & Bradway, 2017; Muntlin, 2018; Richards et al., 2014). When the state of European nursing research is exposed, it shows that research in the leading nursing journals is largely descriptive and poorly described (Richards et al., 2014).

### 4.3 Expertise in advanced nursing

In terms of expertise at the advanced nursing level, development of competencies and skills in the nursing profession should be studied more extensively. With respect to all nurses, measurement and evaluation of competence and skill development should be investigated thoroughly (Harper, Asselin, Kurtz, Macarthur, & Perron, 2012). In addition, factors that support and promote competencies, skills and knowledge evaluation need more attention in current nursing research (Garcia, Cassiani, & Reveiz, 2015; Monterosso, Ross‐Adjie, & Keeney, 2015; Ramelet & Gill, 2012; Wilson, Kelly, Reifsnider, Pipe, & Brumfield, 2013). For example, Harper et al. (2012) and Johnson, Hanvey, Baxter, and Heyland (2013) discovered that educational strategies and teaching methods that support competence development of nurses need to be examined. In particular, the focus ought to be on how skills and competencies are acquired in professional nursing practice and how they can be effectively taught to nurses (Johnson et al., 2013). The focus of nursing research should also be on how to best meet the educational needs of nurses at different stages of development (Ramelet & Gill, 2012).

Effective strategies and models that specialists can use in practice, such as critical thinking and clinical reasoning models, have not been studied enough in nursing science. More research attention than previously granted ought to be put on models for healthcare work processes and inter‐professional collaboration (Garcia et al., 2015). According to multiple researchers, the area of professional development in nursing has to be investigated in greater depth (Harper et al., 2012; Parlour & Slater, 2014; Pillemer et al., 2015; Ramelet et al., 2012). In Monterosso et al.'s (2015) study, respondent nurses identified learning and development as the most important research topics in nursing. While examining nursing and midwifery research priorities, Parlour and Slater (2014) found that career planning, professional appraisal and staff development were considered essential nursing research areas from the nursing experts’ point of view.

### 4.4 Professional nursing practice

The fourth key theme, professional nursing practice, contains three domains where more research is warranted: nursing phenomena, clinical nursing and diseases and specific fields from the nursing perspective.

#### 4.4.1 Nursing phenomena

Regarding the first phenomenon, long‐term care (Brazil, Maitland, Ploeg, & Denton, 2012; Deschodt, Zunica, & Wellens, 2017; Keller, Beck, & Namasivayam, 2015; McGilton et al., 2016; Walsh & Yon, 2012), nursing home settings (Morley et al., 2014; Simmons et al., 2016; Walsh & Yon, 2012), palliative care (Combs, Kluger, & Kutner, 2013; Hanson & Winzelberg, 2013; Henoch et al., 2016; Lunney, 2015; O'Quinn & Giambra, 2014; Ritchie & Zulman, 2013; Schulz, 2013) and basic/fundamental care (Walsh & Yon, 2012) are important phenomena that should be studied more closely. With respect to fundamental care, the practical and emancipatory interests are important to direct nursing research and practice (Granero‐Molina et al., 2018). Moreover, the impact of nursing and organizational models should be one of the research priority areas in long‐term care settings (McGilton et al., 2016).

Improving the quality of life of individuals with chronic conditions (Grady & Gullatte, 2014), patient‐level outcome measures (Davis, Morgans, & Stewart, 2016), patient self‐management (Grady, 2017), personalized health strategies, health promotion and patient education are topics that need further research (Foster et al., 2018; Grady & Gough, 2015). Grady and Gullatte (2014; also Grady, 2017) state that increasing numbers of people are currently living with chronic conditions and managing long‐term illnesses is shifting from health professionals to individuals and their families; consequently, these nursing phenomena ought to be investigated in greater depth. Zwakhalen et al. (2018) proposed a research programme to be developed that aims to create awareness and expand knowledge of evidence‐based basic nursing care by addressing four basic nursing areas: bathing and dressing, communication, mobility and nutrition. They further claim that reassessing these essential nursing activities not only positively influences patient outcomes but has an impact on staff outcomes and organizational outcomes as well (Zwakhalen et al., 2018).

More inquiry is necessary for the fields of midwifery and women's health (Bosco, Williams, Graham, Malagas, & Hauck, 2018; Correa‐de‐Araujo, 2016; Iribarren et al., 2018; Monterosso et al., 2015) and sexual health (Rew, Thurman, & McDonald, 2017). Veteran nursing care (De Jong, 2015; Struwe et al., 2018) and chaplaincy care (Damen, Delaney, & Fitchett, 2018) are also phenomena where research is lacking from the nursing point of view. Furthermore, public health, occupational health and environmental health are considered as fields with a limited number of nursing studies available (Edwards, Porr, & Rieck Buckley, 2015; Issel, Bekemeier, & Kneipp, 2012; McCauley, 2012; Rehfuess et al., 2016). According to Issel et al. (2012), more research ought to be conducted in the area of public health nursing interventions, models and the effectiveness of public health nursing outcomes. Regarding environmental health nursing research, it is recommended that environmental exposures, risk perception, second‐hand smoking and health education on environmental issues need to be the focus of current nursing research (Polivka & Chaudry, 2018). Last, more focus ought to be placed on planning and conducting nursing research in low‐ and middle‐income countries (Rehfuess et al., 2016).

#### 4.4.2 Clinical nursing

Regarding the second domain, nursing research is prioritized focusing on the following clinical areas: paediatric nursing (Brenner et al., 2014; Downing, Knapp, Muckaden, Fowler‐Kerry, & Marston, 2015; Sawin et al., 2012), critical care (Deutschman et al., 2012; Olson et al., 2012), emergency nursing (Hansoti et al., 2017), mental health (Wynaden et al., 2014), oncology nursing (Jarrett et al., 2013; Knobf et al., 2015; Lee, Chung, Chun, Oh, & Cho, 2013; Majidi et al., 2017; Maree, Herbert, & Huiskamp, 2017; Matthews, Danaher Hacker, Otte, & Dean, 2018; Mayer, 2015; Medlow & Patterson, 2015), nephrology nursing (Hewitson, 2014) and medical‐surgical nursing (Davis et al., 2016). In terms of paediatric nursing, the palliative care of paediatric population is one of the topics that needs more attention in nursing research. Children's understanding of death and dying, child and families’ needs assessment and best practices at the end of life are examples of topics that need to be paid attention in nursing science (Brenner et al., 2014; Downing et al., 2015; Ramelet & Gill, 2012). In the Delphi study of mental health nursing (Wynaden et al., 2014), professional issues such as nurses’ job satisfaction and skills and knowledge acquired when working with mental health patients were discovered to be the most important research areas. On the other hand, research should also be focused on clinical issues such as the application of nursing models in mental health (Wynaden et al., 2014). For oncology nursing, research priorities are focusing on clinical issues such as pain management, the identification and relief strategies of symptoms and their associated outcomes (Maree et al., 2017; Mayer, 2015). In addition, living with cancer throughout the trajectory of the disease and cancer prevention interventions should be explored in more detail (Jarrett et al., 2013; Maree et al., 2017).

#### 4.4.3 Diseases and specific fields from the nursing perspective

The third domain encompasses diseases where nursing research is scarce, and as a result, more research is urgently called for. Stroke (Lightbody, 2017; Rowat et al., 2016), heart failure (Stamp et al., 2018), Parkinson's disease (Shin & Habermann, 2016), osteoarthritis (Robbins & Kulesa, 2012), diabetes (Graue et al., 2013; Iversen et al., 2016) and stoma (Hubbard et al., 2017) are considered to be the above‐mentioned areas of diseases. With respect to nursing research on diabetes, Iversen et al. (2016) believe that future research may benefit from larger nurse‐led research programmes organized into networks to share knowledge and expertise across national groups. Autism and developmental disabilities are regarded as specific fields where additional nursing research should be conducted (Tomlinson et al., 2014). Furthermore, wound management and pressure injury (Cowman et al., 2012; Haesler et al., 2018) seem to be specific fields that have a very limited amount of nursing research at the moment. All these areas require more inquiry from the nursing perspective.

## 5 DISCUSSION

In this scoping review, we explored the nursing research priority topics globally. To accomplish this, we identified 91 publications extracted from the CINAHL database that met the inclusion criteria, performed a quality appraisal following the JBI guidelines and analysed the data focusing on research topics of nursing. Overall, four key themes were identified with three additional domains. Here, we highlight a few points of view about the results.

According to our findings, nurse researchers state that a strong emphasis should be put on the development and evaluation of nursing theories. Particularly, middle‐range and situation‐specific theories that link theory to practice need to be generated more actively in nursing science. Some of this concern is well grounded. For example, only a few indications about nursing theory development or nursing theories overall are contained in current nursing research strategies and policy papers (ENRF, n.d.; NINR, 2016). However, there are several textbooks on nursing theories currently available for students and professionals where nursing's theoretical roots are described, different types of theories are presented and their applicability in practice is discussed (Fawcett, 2017; McKenna, Pajnkihar, & Murphy, 2014). As McCarthy and Fitzpatrick (2014, p. xi) point out, nursing as a discipline is rather young and the theoretical development of nursing knowledge is even more recent. Consequently, we need critical dialogue between and among researchers and professionals globally about the current status of theory development in research and nursing practice and how existing theories are evaluated and applied in both contexts. Generally, we ought to have a better understanding how nursing theories are developed and used, particularly in the research of advance knowledge for practice.

Another interesting viewpoint is that long‐term care, palliative care, paediatric nursing and oncology nursing are examples of phenomena where research priorities are actively described whereas only a limited number of publications were found on research priorities for cardiovascular nursing and diabetes nursing even though they are the world's primary causes of disability and death (Driving Sustainable action for Circulatory Health, 2018). There can be several reasons for this imbalance. Using only one database could have impeded exposure to relevant publications about nursing research topics of non‐communicable diseases. Another reason might be that the applied search strategy did not match with the topic, being either too broad or too narrow. Moreover, we did not include grey literature in this review, which might have broadened the scope to more publications on this topic and thereby gave a more complete view of available literature. However, while global research priorities for non‐communicable diseases have been studied from medicine and public health perspectives (Mendis & Alwan, 2011; Sharma, 2017), the number of publications seems to be somewhat limited from the nursing research standpoint, or they are not published in journals that are included in the CINAHL Plus (EBSCO) database.

The current issues in nursing such as patient safety, pain management and professional ethics were not addressed in the publications included in this review. One reason for this might be that in this review, “research priorities” was set as the main search term when the literature search was performed in CINAHL. If researchers have decided not to choose this combination of words/concept to be found in the article or in the keywords, then it is not necessarily included in the results of this literature search.

Different institutions, professional bodies and other stakeholder groups have different future directions for the priorities of nursing research. However, we need to be aware that the profession of nursing itself determines what constitutes the domain of nursing and the research priorities of this domain. Thus, nursing science faculties ought to have more impact when defining research priorities in its own sphere. This review reveals that the topics of nursing research are shattered into pieces, which makes it challenging to have a coherent picture of both current priorities and future directions. Obviously, competitive funding challenges universities and other organizations to create their research profiles and, thus, develop and implement their own research strategies. To strengthen this process, we need effective collaboration among professionals, faculty members and different stakeholders to define mutual top research priorities and to determine clear roles and profiles in nursing research. Moreover, there is a need for additional research to fully evaluate roles, responsibilities, work division and profiles on how nursing research priorities are determined and defined by different nursing actors.

### 5.1 Strengths and limitations

Several researchers suggest that a formal quality assessment of the included publications is not necessary for a scoping review (Munn et al., 2018; Peters et al., 2015; Tricco et al., 2018). Despite this, we applied standardized critical appraisal guidelines (JBI) for the assessment of retrieved publications believing that these actions minimize the opportunity that articles with weak methodological or overall quality were included in the review (Porritt, Gomersall, & Lockwood, 2014). Furthermore, to ensure a more reliable review of included publications, we used an additional research expert to perform the critical appraisal together with one of the authors.

The weakness of this review is that we used one database, CINAHL, for the identification of potentially eligible studies. This was, however, a well‐considered and thoughtful decision as the aim of our review was to analyse and synthetize the research topics of nursing research worldwide and the chosen database is known for being the world's largest source of full‐text nursing and allied health journals at the moment. To minimize this deficiency, the coverage of the literature search was warranted by using the expertise of a library information specialist of health sciences. Second, the lack of hand search of journals might have excluded relevant literature and, in addition, including only English language literature may have limited the findings of this review. One must also note that this review has limited applicability to other contexts because it may not reflect the state of nursing literature in the developing countries. A third restriction would be that, apart from the critical appraisal procedure, only two researchers conducted this review which can be considered as the minimum number of investigators to eliminate the risk of biases.

However, in spite of all the limitations, it is worth pointing out that the data extraction was done systematically and carefully and the data itself, 91 different literature sources, were rich and suitable for thematic analysis method to gain a big‐picture view of current nursing research priorities. After all, the scoping review seeks to present an overview of a potentially large and diverse body of literature pertaining to a broad topic, to identify research gaps and to make recommendations for future research (Arksey & O'Malley, 2005; Armstrong, Hall, Doyle, & Waters, 2011).

### 5.2 Conclusion

Development of nursing theories and usage of robust methods in nursing research were identified as current priorities in nursing science. Expertise in nursing and professional nursing practice with different nursing areas was also recognized as warranting further investigation. These insights can give important information for nursing science faculties to extend the discussion and to strengthen collaboration on nursing research priorities on the local, national and international levels in the future. After all, universities have a special responsibility to produce basic science research, develop and test theories and conduct sound research that generates robust evidence. Based on this review, it appears, however, that the roles are not clear between the professional nursing bodies, research organizations and universities for defining nursing research priorities. It is worth pondering whether professional networks such as the ICN (International Council of Nurses) network for advanced nursing practice could play a significant role in facilitating communication around mutual research interests with universities and, consequently, formulate topics that need to be included in research strategies and action plans.

Nevertheless, research priority setting is a highly essential process and, thus, these results may increase awareness on the current situation of nursing research and will help to identify priority areas that will advance nursing and nursing science for its part in the years to come. Moreover, the results can serve as a basis for the development of nursing research agenda and enable discussion on the status of nursing research.

### 5.3 Recommendations

Based on the results, the following recommendations are suggested. First, a comprehensive systematic review ought to be conducted on nursing research priorities including several relevant databases used in nursing. Since scoping reviews are being used as a precursor to a systematic review, findings of this review can be used as the basis for further systematic review studies. Second, findings of this review can be used as valuable input and resource for nursing research faculties as well as research organizations to form more comprehensive agendas for future research. In addition to faculty research staff, nursing science students may also find these results useful for their studies.

## CONFLICT OF INTEREST

No conflict of interest has been declared by the authors.

## AUTHOR CONTRIBUTIONS

HH and JH: have made substantial contributions to conception and design or acquisition of data, analysis and interpretation of data, gave final approval of the version to be published and agreed to be accountable for all aspects of the work in ensuring that questions related to the accuracy or integrity of any part of the work are appropriately investigated and resolved; HH: involved in drafting the manuscript; JH: revised the manuscript critically for important intellectual content.
